# Sleeping for two: a cross-sectional study on associations between objectively measured sleep during early to mid-pregnancy and maternal and fetal outcomes and inflammatory biomarker profiles

**DOI:** 10.1186/s12884-025-07634-9

**Published:** 2025-05-05

**Authors:** Caitlin Macdonald, Tryfonas Pitsillos, Anna-Karin Wikström, Alkistis Skalkidou, Peter Meerlo, Jocelien Olivier, Jelmer Prins, Inger Sundström Poromaa, Theodora Kunovac Kallak

**Affiliations:** 1 Department of Women’s and Children’s Health, Uppsala University, Uppsala, 751 85 Sweden; 2 Department of Obstetrics and Gynaecology, University of Groningen, University Medical Center Groningen, Groningen, The Netherlands; 3 Neurobiology Expertise Group, Groningen Institute for Evolutionary Life Sciences, University of Groningen, Groningen, The Netherlands

**Keywords:** Sleep quality, Actigraphy, Early pregnancy, Maternal outcomes, Fetal outcomes, Inflammatory biomarkers, Objective sleep parameters

## Abstract

**Background:**

Pregnant women often experience subjective sleep disturbances shown to be associated with maternal and fetal outcomes. However, subjectively experienced sleep often deviates from objective measurements. Therefore, the aim of this study was to explore the relationship between objectively measured sleep in early to mid-pregnancy and maternal and fetal outcomes and inflammatory biomarkers.

**Methodology:**

A total of 1,610 pregnant women aged 18 or older from the Safe Physical Activity in Pregnancy (SPAP) study were recruited during early (week 10–14) to mid-pregnancy (week 16–19). Blood samples were taken and sleep was monitored using an Actiwatch, tracking total sleep time, sleep efficiency, wake after sleep onset, and sleep onset latency for 7 days in early to mid-pregnancy. A combined sleep categorisation was created using total sleep time and sleep efficiency to categorise participants into three sleep quality groups: Good, Intermediate, and Poor. Maternal and fetal outcomes were collected via questionnaires, medical records, and plasma samples were analysed using the Olink cardiovascular paneI Il (*n* = 407).

**Results:**

A total of 1,444 participants were included. The women were categorized as good sleepers (50.4%), intermediate (32.6%), or poor sleepers (17.0%) based on the distribution of the participant’s sleep parameters. Poor sleep was more common in women born outside Europe, those with higher pre-gestational BMI, and those with pre-pregnancy diabetes. Sleep groups did not differ in metabolic factors. Poor sleep was associated with an increased likelihood of requiring an emergency caesarean section (AOR = 1.86, 95% CI 1.13–3.05). No significant associations were found for other outcomes such as pre-eclampsia, premature birth, small for gestational age etc. Nine inflammatory biomarkers were significantly lower in poor sleepers, while one marker was higher.

**Conclusion:**

Poor sleep in early to mid-pregnancy was more common in pregnant women with pre-pregnancy diabetes, obesity, and those born outside of Europe. Poor sleep was associated with a higher likelihood of emergency caesarean section, but no other maternal or fetal outcomes. An overall trend was observed towards lower levels of inflammatory markers in women that slept poorly; however, additional studies are needed to better understand the immune system’s role in the relationship between sleep, maternal health, and maternal and fetal outcomes. Possible mechanisms underlying these associations warrant further research.

**Supplementary Information:**

The online version contains supplementary material available at 10.1186/s12884-025-07634-9.

## Background

Sleep is still an evolutionary conundrum [1, 2], and research is becoming more prolific by the decade as we begin to unravel the mechanisms behind sleep [3–6]. Importantly, it is well established that in general, women report poorer sleep quality and have a higher risk of insomnia than their male counterparts [7, 8]. This discrepancy can be attributable to many factors such as differing reproductive hormones, stress, ageing, depression and perceived responsibilities. In addition, during pregnancy sleep seems even worse and 30% of pregnant women report rarely getting a good night of sleep compared to 15% in the general female population [9–11]. The high rate of perceived sleep problems underlines the importance of sleep research in pregnant women, where physiological and hormonal changes occur by the day and a mother must sleep for two.

A large portion of research has been dedicated to studying sleep in the third trimester of pregnancy, while less can be found on how sleep earlier in pregnancy can affect the mother and/or child. Early pregnancy is a crucial period for fetal development as the fetus experiences rapid growth, the development of major organs and eye and limb movements start to progress [12]. This highlights the importance of research on how maternal sleep during early pregnancy could influence the fetus. Moreover, in early pregnancy poor sleep quality can be due to hormonal changes, nausea, vomiting, itching and back pain but these associations are mostly only based on clinical experiences and require further exploration [13].

While emerging studies address the need for more research on sleep patterns in early pregnancy, many of the studies that address this issue only use subjective sleep measurements such as questionnaires and sleep diaries [13–15]. Subjective sleep measures are often misperceived by the participant and they can deviate substantially from the objective sleep parameters measured by polysomnography or actigraphy [16].

Existing research on sleep disturbances in early pregnancy present associations with various conditions during pregnancy: gestational diabetes [14, 17, 18], obesity [19], blood pressure [20, 21] and depressive symptoms [22]. In terms of pregnancy outcomes, sleep disturbances have been associated with preeclampsia [23], preterm birth [15, 23, 24], perinatal death [15] and caesarean section [25]. The association of sleep disturbance with systemic inflammation has been shown to be associated with postnatal depression [26] and preeclampsia [27]. However, there is still a lack of research on the effect of sleep on the inflammatory biomarkers measured in pregnant women. While many studies suggest sleep disturbances in early pregnancy are associated with poor pregnancy outcomes, Okun et al. contended that as long as the pregnancies were low-risk and healthy, poor sleep does not pose a risk to adverse maternal and fetal outcomes [28]. Again the majority of studies have employed subjective sleep measures [13–15, 17–19, 23–25], and only a few using objective sleep measures [22] or a combination of both [20, 21, 28].

The aim of this study was to determine if there are any associations between objectively measured sleep during early to mid-pregnancy and inflammatory biomarkers and later pregnancy and fetal outcomes, to investigate if poor sleep quality can be a warning sign in maternal healthcare. Our hypothesis was that objectively measured sleep during early to mid-pregnancy would be associated with maternal and fetal outcomes including maternal inflammatory markers, and that maternal sleep should be considered an important component in maternal healthcare.

## Methods and materials

### Participants

Study participants were included in the Safe Physical Activity in Pregnancy (SPAP) study previously described [22], conducted at the Department of Obstetrics, Uppsala University Hospital, Sweden. Between April 2016 and 2023, Swedish speaking women, living in the Uppsala County, were approached for participation in conjunction with their first antenatal ultrasound visit at the Fetal Medicine unit, Uppsala University Hospital. Inclusion was either during the Combined Ultrasound and Biochemical Test appointment (CUB) during week 10 to 14 or at fetal anomaly antenatal ultrasound during week 16 to 19. The Fetal Medicine unit is the only site for antenatal ultrasound, meaning the included women are sampled from the entire population of Uppsala County. Inclusion took place when a research nurse was available, and when equipment was available, i.e. had been returned by prior participants. The inclusion criteria for participants were women above the age of 18, uncomplicated pregnancy at the time of recruitment and being able to wear the Actiwatch during work times (i.e. excluding for example health care staff). Women were expected to wear an Actiwatch for 7 consecutive days at all times. Additional exclusion criterion was only applied for supplementary analyses, where all patients without any available data for inflammatory biomarkers were excluded. All women provided written informed consent to participate in the study after which brief demographic and medical information was obtained, which included items such as ongoing somatic disease, pre-pregnancy smoking, medication, height and weight. The Regional Ethical Review Board in Uppsala, granted ethical approval ([SPAP] 2016/142).

### Sleep

Objective sleep measures were collected by either the Actiwatch-2 or the Actiwatch Spectrum Plus (Philips Respironics, Eindhoven, Netherlands) which sense motion and light. The Actiwatch-2 makes use of piezoelectric accelerometers which detect vertical accelerations within 0.5 and 2.0 g on the wrist and a frequency response range of 0.35–7.5 Hz. The peak accelerations detected over time are used to determine the sleep and wake intervals of the individual. The Actiwatch Spectrum Plus has a different light sensor and a Micromachined micro-electromechanical systems (MEMs) accelerometer, but has the same sampling frequency and their performance in activity recordings and derived sleep statistics are equivalent [29]. Participants were instructed to wear the watch on their non-dominant wrist and stick to their normal weekly routine. They were also required to press the button on the watch to indicate when they went into and got out of bed so that time in bed could be calculated. The Philips Actiware Software version 6.0.9 was used to extract the raw data from the watches. Actograms were visually checked for any malfunctions or failure to check whether participants adhered to the instructions.

The sleep variables measured in this study were Total Sleep Time (TST), Sleep efficiency (SE), Wake After Sleep Onset (WASO), and Sleep onset latency (SOL). These sleep parameters were measured a week after inclusion, either after the CUB appointment between week 10 and 14 or the fetal anomaly antenatal ultrasound during week 16 to 19. The WASO (time awake after falling asleep) and SOL (time taken to fall asleep after getting into bed) were taken to reflect the degree of sleep disruption. The TST was measured as the total duration of sleep in one day in minutes and SE is a ratio of time spent in bed and duration of sleep as a percentage. A combined sleep categorisation was created using TST and SE to categorise participants into three sleep quality groups: Good, Intermediate, and Poor. For these categories, we averaged each women’s TST and SE that slept four days or more and sub-grouped them into tertiles, based on the distribution of these sleep parameters in the cohort. Women in the Poor sleep quality category were in the lowest tertile of both TST and SE (i.e. having both low TST and low SE), women in the Good sleep quality category were women with either high TST or SE or in the highest tertiles of both TST and SE, and remaining women, were categorized as having Intermediate sleep quality. The TST tertile cut-offs were: Low ($\:\le\:$ 415.00 min), intermediate (415.01–458.99 min) and high ($\:\ge\:$459.00 min). The SE tertile cut-offs were: Low ($\:\le\:$81.21%), intermediate (81.22–85.26%) and high ($\:\ge\:$85.27%). The aim of this combined sleep categorisation was to create an exposure variable that would encapsulate the quality of sleep on all fronts. Sleep quality is characterised by TST, SE, WASO and SOL [30], however we have only included TST and SE as WASO and SOL are also reflected in SE, by illustrating missed sleep due to difficulty to fall asleep and not wake during sleep in the overall percentage. Moreover, by combining these parameters into one variable, we decrease the number of statistical tests needed and also the risk of false positive significance.

### Study protocol and outcomes

At enrolment of this cross-sectional study, participants completed a questionnaire providing information on demographics, current health and health behaviors, as previously described for the SPAP study [22]. Data was obtained on age, parity, pre-pregnancy smoking, ongoing somatic and mental health condition and medication. In the course of the study, information on maternal characteristics was supplemented with data from the antenatal and delivery medical records, providing metabolic variables and delivery and fetal outcomes. Pre-gestational weight and height were self-reported and collected at inclusion of the study and pre-gestational Body Mass Index (BMI) calculated. When categorising BMI, the following cut-offs were employed; underweight (BMI = < 18.5), normal (BMI = 18.5–24.9), overweight (BMI = 25.0-29.9), obese (BMI = 30.0–35.0) and severely obese (BMI = > 35). Delivery mode was also categorized into planned caesarean section, spontaneous vaginal delivery, vacuum extraction, and emergency caesarean section (emergency or acute), with cases that were initially recorded as a planned caesarean section being excluded from the latter three categories when coding the variables in the database. The emergency Caesarean section group includes both acute C-section, when during labour a natural delivery is no longer possible, and emergency, when immediate action is needed in a life threatening situation.

The metabolic and physiologic factors measured include weight increase, change in systolic and diastolic blood pressure, the highest glucose measurement and the lowest haemoglobin measurement. The weight increase is the weight gained from the self-reported pre-gestational weight to the final weight measurement at birth. Change in systolic and diastolic blood pressure measures the difference in blood pressure of the mother between the first trimester and before birth. The glucose measurements were taken from non-fasting capillary samples at midwife appointments, whereby the highest measurement was chosen. Similarly, haemoglobin measurements were measured with capillary samples at the same midwife appointments.

The birth weights were standardised according to the national reference, based on the gestational age and sex of the child [31, 32]. Small for gestational age (SGA) was defined as a standardised weight after birth in the lower 10th percentile and Large for gestational age (LGA) in the upper 90th percentile for a healthy baby born with no conditions or abnormalities. Other categorised variables included: season winter (November- February) or other time of the year, psychiatric condition (any ongoing mental health condition and/or use of psychiatric medication as reported at the first antenatal booking), preterm birth (< week 37), postterm birth (> week 42) and Fetal growth restriction (FGR) (Estimated fetal weight in utero < tenth percentile for gestational age, most often due to a condition or abnormality). Protein analysis was collected through the Olink cardiovascular II panel, which measures 92 blood-based biomarkers that are known or suspected to be markers of cardiovascular and inflammatory disease [33]. Blood samples were taken during the first appointment after agreeing to enrol, this could either be the CUB appointment between week 10 and 14 or the fetal anomaly antenatal ultrasound during week 16 to 19. The specific time of day at which the blood sample was taken is unknown, but usually between 8 am to 4 pm. The season in which the measurement took place was either Winter (November-February) or outside of that. Since the study took place in Sweden where daylight during Winter is short compared to most other European countries we wanted to explore any possible association. Since season has been shown to be associated with sleep [34] and inflammatory markers [35].

### Statistics

Descriptive statistics were employed to present group comparisons of population characteristics, examining various factors including demographic data to identify confounders when analysing metabolic, pregnancy, birth, and fetal outcomes across the three sleep quality groups (Good, Intermediate, and Poor). Comparison of means within the groups of continuous variables was done by Analysis of variance (ANOVA) tests, with the inflammatory biomarkers that differed significantly, additional Bonferroni post-hoc tests were performed to determine which specific sleep category differed significantly. The same was calculated through Chi-square tests for categorical variables and corresponding Bonferroni adjustment post-hoc tests. Ordinal logistic regression was used to estimate the association between sleep and pregnancy, birth and fetal outcomes. Crude odd ratio (COR) and adjusted odds ratio (AOR) was found with their respective 95% confidence intervals. A generalised linear model was used to analyse the association between the sleep groups and the inflammatory biomarkers in the Olink panel that had significant results after an ANOVA. All models were adjusted for the same significant confounders; country of birth, calculated pre-gestational BMI, pre-pregnancy diabetes and parity. We selected a minimum set of four constant confounders for each logistic regression to ensure relevance across all outcomes. To assess collinearity among our confounders in the final models, we calculated the variance inflation factors (VIFs), ensuring that all VIF values were below 1.5, indicating no evidence of collinearity. All statistical analyses were done using IBM SPSS Statistics version 29.0.2.0.

## Results

### Group characteristics and inclusion

**Fig. 1:**
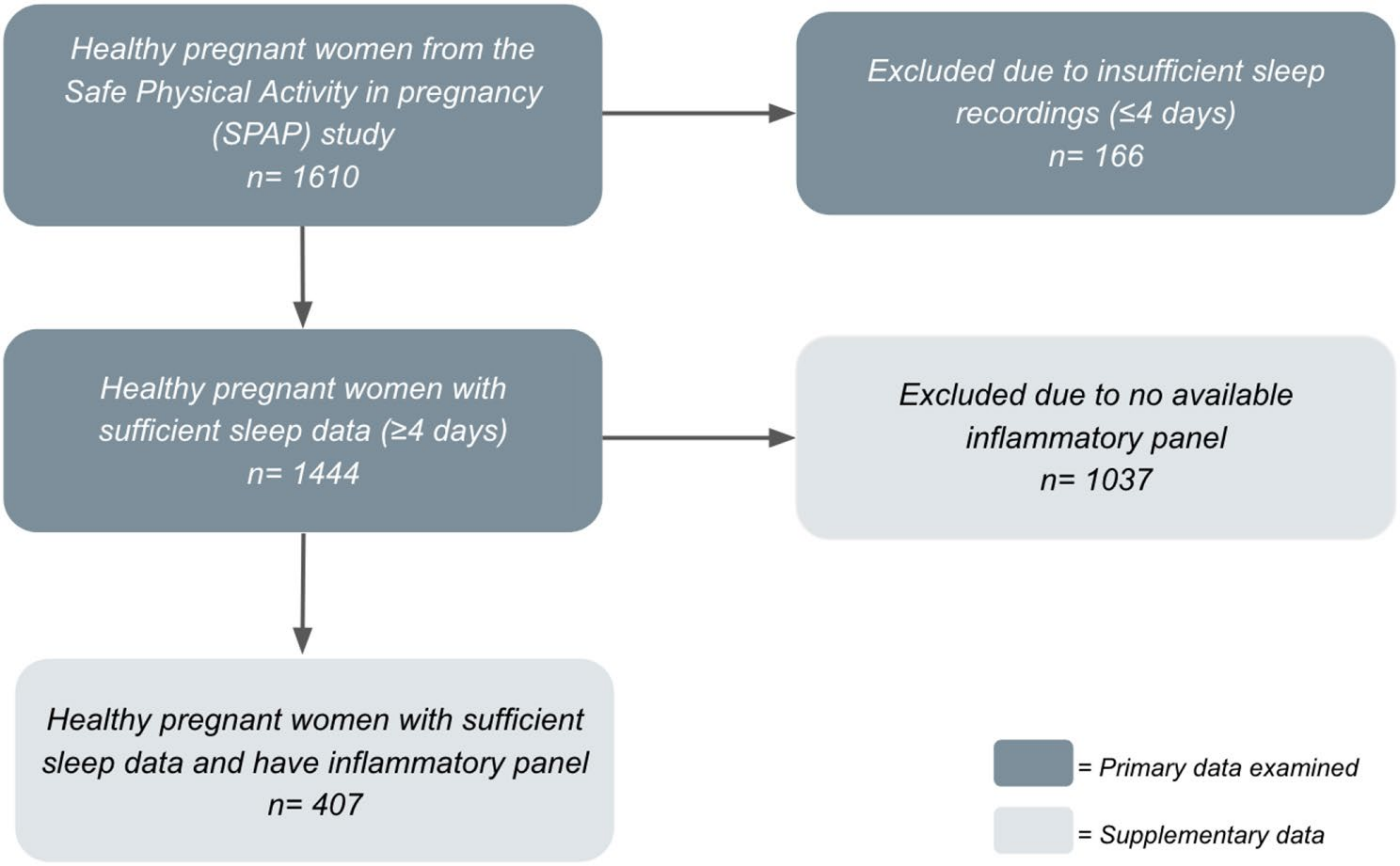
Flow chart depicting population selection using inclusion and exclusion criteria [Additional File 1]

After excluding all women who had fewer than four days of data recorded on the Actiwatch, a total of 1,444 participants remained for analysis (Fig. 1). The sleep characteristics of the study population, measured at a mean week of 14.7 ± 3.0, were as follows; the average TST was 7.3 ± 1.0 h (good *n* = 728, intermediate *n* = 471 and poor *n* = 245) and the average sleep efficiency was 82.4 ± 5.9% (good *n* = 727, intermediate *n* = 471, poor *n* = 245). In terms of sleep disruption, women had an average WASO of 53.9 ± 23.1 min per night and it took an average of 23.4 ± 19.4 min for women to fall asleep. As shown in Tables 1 and 50.4% of women had good sleep quality, 32.6% had intermediate sleep quality and 17.0% poor had poor sleep quality. Furthermore Table 1 shows the remaining study characteristics including confounders for the outcomes presented later in Tables 2, 3, 4 and 5.

**Table 1:** Group comparison of the population characteristics

		Good sleep *N* = 728 (50.4%)		Intermediate sleep *N*= 471 (32.6%)		Poor sleep *N* = 245 (17.0%)		
Variable		N	Mean ± SD	N	Mean ± SD	N	Mean ± SD	P-value a
**Age (years)** *N* = 1443		728	31.4 ± 4.4	470	31.4 ± 4.5	245	32.0 ± 4.8	0.186
**Gestational week of assessment** *N* = 1423	718		14.7 ± 2.9	461	14.9 ± 3.0	244	14.4 ± 3.1	0.125
**Parity** *N* = 1431								
* Nulliparous*		373 (51.6%)		223 (48.1%)		107 (43.9%)		0.096
* Multiparous*		350 (48.4%)		241 (51.9%)		137 (56.1%)		
**Country of birth** *N* = 1444								
* Sweden and Europe*		675 (92.7%)		426 (90.4%)		207 (84.5%)*		**< 0.001**
* Outside Europe*		53 (7.3%)		45 (9.6%)		38 (15.5%)*		
**First trimester in winter** *N* = 1444		403 (55.4%)		250 (53.1%)		126 (51.4%)		0.509
**Pre-gestational weight (kg)** *N* = 1424		720	69.5 ± 12.6	464	70.2 ± 15.6	240	69.8 ± 14.2	0.636
**Pre-gestational BMI (**$\:{kg/m}^{2}$**)** *N* = 1424								
* Underweight*		10 (1.4%)		16 (3.4%)		3 (1.3%)		**< 0.001**
* Normal weight*		429 (59.6%)		253 (54.5%)		148 (61.7%)		
* Overweight*		197 (27.4%)		127 (27.4%)		49 (20.4%)		
* Obese*		71 (9.9%)		47 (10.1%)		24 (10.0%)		
* Severely obese*		13 (1.8%)		21 (4.5%)*		16 (6.7%)*		
**Smoker** *N* = 1430		57 (7.9%)		36 (7.8%)		20 (8.2%)		0.976
**Systolic BP 1st trimester** *N* = 1393 **(mmHg)**		709	113.1 ± 9.8	450	113.1 ± 9.8	234	112.5 ± 10.9	0.631
**Diastolic BP 1st trimester** *N* = 1391 **(mmHg)**		707	68.7 ± 8.7	450	68.4 ± 8.7	234	67.6 ± 8.8	0.240
**Pre-pregnancy diabetes** *N* = 1435		4 (0.6%)		5 (1.1%)		6 (2.5%) *		**0.040**
**Pre-pregnancy hypertension** *N* = 1435		7 (1.0%)		7 (1.5%)		4 (1.6%)		0.606
**Psychiatric condition or medication** *N* = 1426		96 (13.3%)		70 (15.1%)		45 (18.6%)		0.134
**Sleep during pregnancy compared to before** *N* = 1047								
* Better*		27 (5.0%)		27 (8.4%)		9 (4.9%)		0.318
* Equal*		243 (45.0%)		137 (42.4%)		80 (43.5%)		
* Worse*		270 (50.0%)		159 (49.2%)		95 (51.6%)		

N = number, SD = standard deviation, BMI = body mass index, BP = blood pressure

*Significantly different from good sleep

✝ Significantly different from intermediate sleep

a Calculated using ANOVA or Chi-square fisher’s exact test

**Table 2:** Group comparison of the metabolic and physiological indicators

	Good sleep *N* = 728 (50.4%)		Intermediate sleep *N*= 471 (32.6%)		Poor sleep *N* = 245 (17.0%)		
Variable	N	Mean ± SD	N	Mean ± SD	N	Mean ± SD	P-value a
Weight increase [pregestational-final] (kg) *N* = 936	451	13.6 ± 5.0	311	13.2 ± 4.9	174	12.9 ± 13.3	0.263
Change in systolic BP [1st-final] (mmHg) *N* = 1100	530	6.7 ± 11.8	365	7.2 ± 12.0	205	6.2 ± 15.0	0.655
Change in diastolic BP[1st-final] (mmHg) *N* = 1100	530	7.1 ± 10.7	365	6.5 ± 10.6	205	7.4 ± 11.0	0.535
Highest glucose measurement (mmol/L) *N* = 965	447	6.0 ± 1.0	326	6.0 ± 1.0	192	6.0 ± 1.1	0.961
Lowest HB measurement (g/L) *N* = 974	451	113.5 ± 9.9	328	112.7 ± 9.4	195	113.2 ± 9.7	0.481

N = number, SD = standard deviation, BMI = body mass index, BP = blood pressure, 1st = first trimester, final = final measurement before birth, HB = haemoglobin

a Calculated using ANOVA

**Table 3:** Ordinal logistic regression of sleep groups for pregnancy, birth and fetal outcomes

	Good sleep *N* = 728 (50.4%)		Intermediate sleep *N*= 471 (32.6%)			Poor sleep *N* = 245 (17.0%)		
**Variable**	**N**	**Ref**	**N**	**Crude OR (95% CI)**	**Adjusted OR (95% CI)**	**N**	**Crude OR (95% CI)**	**Adjusted OR (95% CI)**
**Preeclampsia** *N* = 1376	25 (3.6%)		23 (5.2%)	1.46 (0.82–2.61)	1.41 (0.78–2.54)	12 (5.2%)	1.47 (0.73–297)	1.44 (0.69–3.01)
**Gestational diabetes** *N* = 1376	20 (2.9%)		16 (3.6%)	1.26 (0.65–2.46)	1.06 (0.53–2.11)	6 (2.6%)	0.90 (0.36–2.27)	0.74 (0.28–1.93)
**Gestational hypertension** *N* = 1376	29 (4.2%)		12 (2.7%)	0.64 (0.32–1.26)	0.64 (0.32–1.27)	12 (5.2%)	1.26 (0.63–2.51)	1.41 (0.69–2.87)
**Preterm birth** *N* = 1366	33 (4.8%)		32 (7.2%)	1.56 (0.94–2.57)	1.51 (0.90–2.51	15 (6.5%)	1.38 (0.74–2.59)	1.22 (0.63–2.38)
**Postterm birth** *N* = 1380	185 (26.4%)		107 (23.9%)	0.88 (0.67–1.15)	0.89 (0.67–1.18)	46 (19.7%)	0.69* (0.48–0.99)	0.76 (0.52–1.10)
**Labour dystocia** *N* = 1360	120 (17.4%)		69 (15.7%)	0.89 (0.64–1.22)	0.94 (0.67–1.31)	34 (14.7%)	0.82 (0.54–1.24)	0.92 (0.59–1.42)
**Mode of delivery** *N* = 1376								
* Vaginal*	558 (79.9%)		337 (75.6%)	0.87 (0.63–1.21)	0.86 (0.61–1.21)	171 (73.7%)	0.80 (0.53–1.19)	0.70 (0.46–1.08)
* Vacuum extraction*	47 (6.7%)		23 (5.2%)	0.78 (0.47–1.31)	0.85 (0.50–1.44)	11 (4.7%)	0.72 (0.37–1.41)	0.81 (0.40–1.65)
* Planned CS*	36 (5.2%)		37 (8.3%)	1.66* (1.03–2.68)	1.59 (0.98–2.57)	21 (9.1%)	1.83* (1.05–3.20)	1.67 (0.93–3.01)
* Acute and emergency CS*	57 (8.1%)		49 (11.0%)	2.45 (0.97–2.16)	1.42 (0.94–2.15)	29 (12.5%)	1.69* (1.05–2.72)	1.86* (1.13–3.05)
**Induction of birth** *N* = 1283	169 (25.5%)		115 (28.1%)	1.14 (0.87–1.51)	1.12 (0.84–1.49)	56 (26.4%)	1.05 (0.74–1.49)	1.08 (0.74–1.55)
**Standardisation birth weight** *N* = 1364								
* SGA*	31 (4.5%)		29 (6.5%)	1.48 (0.88–2.48)	1.58 (0.93–2.68)	13 (6.6%)	1.26 (0.65–2.45)	1.56 (0.79–3.08)
* AGA*	571 (83.1%)		346 (78.1%)	0.76 (0.58-1.00)	0.77 (0.58–1.02)	186 (80.5%)	0.87 (0.62–1.22)	0.90 (0.62–1.29)
* LGA*	85 (12.3%)		68 (15.3%)	1.28 (0.91–1.80)	1.27 (0.89–1.81)	32 (13.9%)	1.14 (0.74–1.76)	1.20 (0.69–1.74)
**NICU stay** *N* = 1375	80 (11.5%)		58 (13.0%)	1.51 (0.80–1.65)	1.12 (0.77–1.62)	25 (10.7%)	0.93 (0.58–1.49)	0.81 (0.49–1.34)
**FGR** *N* = 1376	4 (0.5%)		5 (1.1%)	1.97 (0.53–7.37)	2.64 (0.66–10.67)	3 (1.2%)	2.27 (0.51–10.23)	4.06 (0.82–20.14)
**IUFD** *N* = 1376	2 (0.3%)		4 (0.8%)	3.15 (0.57–17.27)	2.80 (0.49–15.95)	1 (0.4%)	1.51 (0.14–16.69)	1.07 (0.9-13.27)

N = number, OR = odds ratio, CI = confidence interval, CS = C-section, SGA = small for gestational, AGA = appropriate for gestational age, LGA = large for gestational age, NICU = neonatal intensive care unit, FGR = fetal growth restriction, IUFD = intrauterine fetal death. Variables adjusted for: Country of birth, BMI at first antenatal visit, Pre-pregnancy diabetes and Parity

*Significantly different from good sleep

**Table 4:** Group comparison of the inflammatory variables measured during week 18

	Good sleep *N* = 728 (50.4%)		Intermediate sleep *N*= 471 (32.6%)		Poor sleep *N* = 245 (17.0%)		
**Variable** *N* = 407	**N**	**Mean ± SD**	**N**	**Mean ± SD**	**N**	**Mean ± SD**	**P-value** a
SRC	196	6.25 ± 0.93	146	5.91 ± 0.91*	65	5.88 ± 0.98*	< 0.001
CD40L	196	4.07 ± 1.09	146	3.76 ± 0.80*	65	3.70 ± 0.98*	0.004
STK4	196	3.87 ± 1.01	146	3.58 ± 0.46*	65	3.52 ± 0.97*	0.007
THPO	196	2.68 ± 0.31	146	2.63 ± 0.29	65	2.77 ± 0.50*	0.026
DECR1	196	4.06 ± 0.95	146	3.72 ± 0.88*	65	3.78 ± 0.90	0.002
ITGB1BP2	196	3.48 ± 1.18	146	3.14 ± 0.87*	65	3.13 ± 1.09	0.006
NEMO	196	4.35 ± 0.87	146	4.11 ± 0.73*	65	4.09 ± 0.82	0.009
HSP27	196	9.63 ± 0.46	146	9.50 ± 0.49 *	65	9.50 ± 0.49	0.019
IL27	196	7.65 ± 0.30	146	7.56 ± 0.32*	65	7.61 ± 0.32	0.024

N = number, SCR = Proto-oncogene tyrosine-protein kinase, CD40L = CD40 ligand, STK4 = Serine/threonine-protein kinase 4, THPO = thrombopoietin, DECR1 = 2,4-dienoyl-CoA reductase 1, ITGB1BP2 = Integrin Subunit Beta 1 Binding Protein 2, NEMO = nuclear factor κB (NF-κB) essential modulator, HSP27 = Heat shock protein-27, IL27 = Interleukin 27

*Significantly different from good sleep

* Significantly different from intermediate sleep

a Calculated using ANOVA

**Table 5:** Adjusted univariate generalised linear model of sleep quality and inflammatory biomarkers

	Good sleep *N* = 728 (50.4%)		Intermediate sleep *N*= 471 (32.6%)					Poor sleep *N* = 245 (17.0%)				
**Variable** *N* = 407	**N**	**Ref**	**N**	**βa (95% CI)**	**Std error**	**t**	**p**	**N**	**βa (95% CI)**	**Std error**	**t**	**p**
SRC	196		146	**-0.34 (-0.54**, -0.14)*	**0.10**	**-3.36**	**< 0.001**	65	**-0.40 (-0.66**, -0.14)*	**0.13**	**-3.00**	**0.003**
CD40L	196		146	**-0.31 (-0.52**, -0.10)*	**0.11**	**-2.93**	**0.004**	65	**-0.40 (-0.67**, -0.12)*	**0.14**	**-2.82**	**0.005**
STK4	196		146	**-0.29 (-0.50**, -0.08)*	**0.11**	**-2.69**	**0.007**	65	**-0.37 (-0.65**, -0.10)*	**0.14**	**-2.66**	**0.008**
THPO	196		146	-0.05 (-0.12, 0.03)	0.04	-1.28	0.201	65	0.09 (-0.00, 0.19)	0.05	1.92	0.055
DECR1	196		146	**-0.35 (-0.55**, -0.15)*	**0.10**	**-3.49**	**< 0.001**	65	**-0.31 (-0.57**, -0.05)*	**0.13**	**-2.37**	**0.018**
ITGB1BP2	196		146	**-0.34 (-0.57**, -0.11)*	**0.12**	**-2.93**	**0.004**	65	**-0.37 (-0.67**, -0.07)*	**0.15**	**-2.41**	**0.016**
NEMO	196		146	**-0.25 (-0.43**, -0.08)*	**0.09**	**-2.86**	**0.004**	65	**-0.29 (-0.52**, -0.06)*	**0.12**	**-2.49**	**0.013**
HSP27	196		146	**-0.13 (-0.23**, -0.03)*	**0.05**	**-2.54**	**0.011**	65	**-0.14 (-0.28**, -0.01)*	**0.07**	**-2.10**	**0.037**
IL27	196		146	**-0.09 (-0.16**, -0.03)*	**0.03**	**-2.73**	**0.007**	65	-0.03 (-0.11, 0.06)	0.05	-0.58	0.566

N = number, Ref = reference group, βa = adjusted beta coefficient, Std = standard, t = t-value, p = p-value, SCR = Proto-oncogene tyrosine-protein kinase, CD40L = CD40 ligand, STK4 = Serine/threonine-protein kinase 4, THPO = thrombopoietin, DECR1 = 2,4-dienoyl-CoA reductase 1, ITGB1BP2 = Integrin Subunit Beta 1 Binding Protein 2, NEMO = nuclear factor κB (NF-κB) essential modulator, HSP27 = Heat shock protein-27, IL27 = Interleukin 27. Variables adjusted for: Country of birth, BMI at first antenatal visit, Pre-pregnancy diabetes and Parity

*Significantly different from good sleep

Country of birth, calculated pre-gestational BMI, and pre-pregnancy diabetes differed between the sleep groups (p = < 0.001, p = < 0.001, *p* = 0.040 respectively). Women born outside of Europe were more frequently classified as poor sleepers than intermediate or good compared to those born in Sweden or Europe (*p* = 0.028 and p = < 0.001 respectively). Additionally, there were more severely obese women who had poor or intermediate sleep quality (*p* = 0.019 and p = < 0.0001). Lastly, more women with pre-pregnancy diabetes had poor sleep quality (*p* = 0.019). Otherwise, we found no differences in baseline characteristics.

### Physiological and metabolic factors

To analyse the effects of early to mid-pregnancy sleep patterns on metabolic and physiological indicators throughout gestation, a group comparison of metabolic and physiological outcomes was performed for each sleep category (Table 2). There were no significant differences in metabolic factors or physiological indicators among the different sleep categories. Missing data was due to discrepancies in how medical information was recorded in the electronic patient-database, i.e. if a value was added as a note on a patient file instead a numeric value where indicated.

### Pregnancy and fetal outcomes

To examine the relationship between sleep quality in early to mid-pregnancy and a range of pregnancy and fetal outcomes an ordinal logistic regression analysis was performed to obtain the crude and adjusted odd ratios (Table 3). Some crude associations were noted for women in the Poor sleep quality category, such as lower risk of postterm birth and increased risk of planned caesarean section as mode of delivery, however, none of these remained following adjustment. However, poor sleep quality in early to mid-pregnancy increased the odds of women requiring an emergency caesarean section (COR = 1.69, 95% CI = 1.05–2.72). After adjusting the model for country of birth, pre-gestational BMI, pre-pregnancy diabetes and parity, the odds remained significant (AOR = 1.86, 95% CI = 1.13–3.05). Apart from these findings, no further significant associations were identified for increased odds of experiencing major pregnancy complications, adverse birth outcomes, or negative fetal outcomes between the sleep groups.

### Inflammatory biomarkers

To analyse the association of inflammatory values with different sleep categories a general linear model was performed. The following inflammatory values showed significant differences among sleep categories after ANOVA and Bonferroni tests: Proto-oncogene tyrosine-protein kinase (SRC), CD40 ligand (CD40L), Serine/threonine-protein kinase 4 (STK4), Thrombopoietin (THPO), 2,4-dienoyl-CoA reductase 1 (DECR1), Integrin Subunit Beta 1 Binding Protein 2 (ITGB1BP2), Nuclear factor κB (NF-κB) essential modulator (NEMO), Heat shock protein-27 (HSP27), Interleukin 27 (IL27) (Table 4). Generally, levels of inflammatory markers were lower with poor sleep quality, apart from THPO which showed higher levels with poor sleep. Supplementary Table 1 includes a group presentation of the remaining inflammatory markers that showed no significant differences across the sleep groups [Supplementary data file].

Table 5 shows the adjusted linear regression models of the sleep categories and inflammatory biomarkers. All inflammatory biomarkers (SRC, CD40L, STK4, DECR1, ITGB1BP2, NEMO, HSP27, IL27) show significant lower levels of biomarkers in intermediate sleepers compared to good, apart from THPO which now has an insignificant difference among the sleep categories. Similarly, poor sleepers show the same trend however IL27 also has no significantly lower levels compared to good sleepers. It can be noted however that HSP27, DECR1, ITGB1BP2 and NEMO show a low beta coefficient after being adjusted for poor sleep.

## Discussion

### Main findings

The main finding of this study was that poor sleep during early to mid-pregnancy is not associated with any major adverse pregnancy, delivery or fetal outcomes apart from an increased risk of emergency Caesarean section. This may in part be due to the fact that in our cohort most women slept reasonably well and showed modest variation in sleep quality. Additionally, women who were born outside of Europe, severely obese or had pre-existing diabetes were more often categorized as having poor sleep quality. In general, levels of inflammatory biomarkers were lower in mothers in the poor or intermediate sleep quality categories compared to those in the good sleep quality group.

### Interpretation

Our study found that women with pre-existing diabetes and severe obesity more often had poor sleep quality, consistent with existing literature linking these factors to sleep disturbances [36]. However, research on the effect of pre-existing diabetes and obesity on poor sleep in pregnancy is limited, with more focus on how sleep disturbances contribute to gestational diabetes, elevated BMI, and high blood pressure [14, 18, 21, 37, 38]. Morselli et al. refer to this as “diabesity,” where sleep loss impairs insulin sensitivity, increases appetite, and contributes to obesity and diabetes. Despite this, our analysis did not find an association between poor sleep and these maternal conditions later in pregnancy. A reason for this could be the change in Swedish diagnostic criteria for gestational diabetes during a few months in 2018 [39]. However, this should only affect the power to detect the association as all sleep groups were affected by this change. Another reason could be due to differences in study population regarding sleep characteristics, since women in our cohort slept relatively good, previous studies might have study populations with a poorer sleep quality leading to the discrepancy in association with gestational diabetes.

Furthermore, our analyses show that non-European women experience worse sleep quality. Literature indicates that African American pregnant women sleep worse than Caucasian women [40], but other studies find similar rates of sleep complaints among African American, Hispanic, Latino, and Asian groups compared to Caucasians [41]. Additionally, lower socioeconomic status is linked to higher rates of sleep complaints, highlighting its role as a potential confounder [41]. These conflicting findings underscore the complexity of the issue and suggest that factors like geographical location and study design may influence outcomes. Overall, further investigation is needed to understand the factors affecting sleep quality among different racial and ethnic groups.

After adjustment, women with poor sleep quality in early to mid-pregnancy exhibited higher odds of having an emergency Caesarean section, consistent with existing literature. Studies indicate that poor sleep increases the risk of a Caesarean section in general [25, 42, 43], a planned Caesarean Sect. [44] or an emergency Caesarean section in nulliparous women [45]. Before adjustment to the ordinal logistic regression, our analysis also showed higher odds of a planned Caesarean section among women in the poor and intermediate sleep quality categories. The lack of significant association after adjustment in our study may be due to various factors, including the reliance on subjectively measured sleep parameters in most other studies, potentially limiting the exposure variable’s sensitivity [25, 42, 44, 45]. Additionally, we adjusted the regression for pre-gestational BMI, parity and pre-pregnancy diabetes, all factors that are known to affect the incidence of planned caesarean Sects. [46, 47].

Over the past years research has provided more evidence on how sleep in non-pregnant individuals enhances the immune defence, whereby different mechanisms and signalling pathways play a role [48]. During pregnancy, the body’s innate immune responses are physiologically regulated to prevent the rejection of the fetal allograft, with key adaptations involving changes in cytokine production [49]. During normal early-mid pregnancy, oestrogen and regulatory proteins work together to modulate decidual stromal cells, dendritic cells, Treg cells, NK cells, and effector T cells, promoting a state of pregnancy tolerance characterized by elevated IL-10 and other anti-inflammatory cytokines. The pro-inflammatory environment which was heightened during implantation and placentation becomes more suppressed [50]. In contrast to that during physiological sleep, mainly pro-inflammatory and/or Th1 cytokines peak [51]. Our hypothesis was that poor sleep may be associated with alterations in inflammatory markers, our analysis identified significant differences in the levels of inflammatory markers such as SRC, CD40L, STK4, THPO, DECR1, ITGB1BP2, NEMO, HSP27, and IL-27 between women in the poor/intermediate sleep categories and good sleepers. None of these biomarkers are pro-inflammatory, anti-inflammatory or Th1 cytokines, apart from the pleiotropic cytokine IL27 that has both pro and anti-inflammatory properties [52, 53], which after adjustment did not appear significantly different with regard to sleep. Furthermore during the active wake period, as a result of different forms of cellular stress such as physical activity, metabolism and cell injury an accumulation of factors that resemble exogenous danger signals, will trigger the aforementioned peak of cytokines during sleep [51]. One of these factors includes heat shock proteins (HSP). It can be hypothesised that a longer active wake period in participants with poor sleep quality would lead to higher HSP27 levels, however our study showed lower levels in this group compared to those in the good sleep quality category. Reasons for this could be that the majority of poor sleepers in our study slept worse due to a higher WASO. Alternatively, it’s likely that due to our study population sleeping relatively well in terms of duration and efficiency, they did not produce a stress response and subsequent release of cytokines that have been associated with sleep in existing literature [54–56]. It is also a limitation that blood samples were taken during working hours and not strictly at a specific time point after sleeping. In general, observing the impact of disturbed sleep on immune parameters is challenging because the immune system is highly dynamic, and since we only have a measurement from a single time-point this could explain our opposing results.

As previously noted, in our study most major pregnancy complications were not associated with poor sleep in early to mid-pregnancy. However, a recent large prospective cohort study involving 2,703 women found that poor sleep quality was associated with an increased risk of gestational diabetes mellitus, preterm delivery, and perinatal death [15]. Other studies have also reported correlations between poor sleep and conditions such as preeclampsia, preterm birth, and premature rupture of membranes (*n* = 500) [23]. Again, the discrepancies between these findings and ours may be attributed to factors such as smaller sample sizes, reliance on subjectively measured sleep data, or the use of less comprehensive sleep parameters as exposure variables.

### Strengths and limitations

This study had both strengths and limitations. One of the major strengths of this study was the objectively measured sleeping parameters. The golden standard for sleep measurements is polysomnography; however, research has shown minimal differences compared to actigraphy [57, 58]. Due to its affordability and portability, actigraphy is recommended over polysomnography for measuring sleep in home settings [59]. Furthermore, this study analysed a sizeable population, excluding only a minimal number of cases to preserve generalisability.

Some limitations included issues measuring sleep. Firstly, there were two types of Actiwatches used to collect the data in the study, both having slightly different mechanisms to measure sleep. It is a limitation that this study was not able to control for the two types of actigraph watches, however the manufacturer states that these two devices are equivalent [29] and therefore it should not affect the results of the study. This assumption has also been made in a previous comparison with polysomnography [60]. Furthermore, our analysis was based on the distribution of TST and SE for grouping into sleep categories, based on tertiles within the study cohort, and in general our participants slept well, therefore, one of the major limitations of this study is the small difference in sleep between good and poor sleep quality categories. Additionally, although participants were questioned whether they slept better, equally or worse during pregnancy compared to before pregnancy (see Table 1), our study lacks subjective sleep parameters. Sleep is considered to be multidimensional with components such as satisfaction and alertness that only subjective sleep measures can provide [61]. In the future we would record sleep with a combination of both objective and subjective sleep parameters. Moreover, early pregnancy related complaints such as nausea, vomiting, nycturia and pain vary by women, which could have a confounding effect on sleep. Concerning confounding factors, there are a few unmeasured confounding variables not accounted for in this study, including the aforementioned early pregnancy complaints. Factors such as socioeconomic status, occupation, education and stress can have a major effect on sleep [41, 62] but were not captured in this study. Furthermore, our database did not include any pre-existing sleep disorders, due to there being no mention of sleep disorders in their self-reported conditions or medication. This is important as it could explain our associations between pre-existing diabetes, obesity and sleep. Additionally, the cross-sectional design, where blood was only collected at one time point and later used for analysis of inflammatory markers, hinders any analysis regarding changes of inflammatory markers over time. Lastly, many of the outcomes in the results had a limited number of affected women for outcomes such as preeclampsia, gestational diabetes, SGA, FGR, and IUFD. This could lead to insufficient statistical power for reliable results for outcomes like SGA and FGR in particular.

## Conclusion

Poor sleep quality in early to mid-pregnancy appears to increase the likelihood of an emergency Caesarean section, but does not affect the risk for other pregnancy complications. Poor sleep could therefore be seen as a possible warning sign for an emergency delivery but more research is needed to strengthen this claim. Additionally, factors such as obesity, pre-existing diabetes, and non-European origin are linked to poor sleep quality, emphasising the need for targeted interventions. The relationship between sleep and inflammatory biomarkers during pregnancy requires further exploration. This study underscores the importance of improving the recording of sleep measurements and addressing confounders in future research.

## Electronic supplementary material

Below is the link to the electronic supplementary material.

Supplementary Material 1

## Data Availability

The datasets generated and/or analyzed during the current study are not publicly available due restrictions based on the Swedish Ethical Review Act (2003:460) and sensitive date protection regulated by the General Data Protection Regulation (2016:679). Data can be made available from the corresponding author on reasonable request including personal data processing agreements.

## Abbreviations

SPAP: Safe Physical Activity in Pregnancy
CUB: Combined Ultrasound and Biochemical Test appointment
MEMs: Micromachined micro-electromechanical systems
TST: Total Sleep Time
SE: Sleep efficiency
WASO: Wake After Sleep Onset
SOL: Sleep onset latency
BMI: Body Mass Index
C-section: Caesarean section
SGA: Small for gestational age
LGA: Large for gestational age
FGR: Fetal growth restriction
ANOVA: Analysis of variance
COR: Crude odd ratio
AOR: Adjusted odds ratio
VIF: Variance inflation factor
SRC: Proto-oncogene tyrosine-protein kinase
CD40L: CD40 ligand
STK4: Serine/threonine-protein kinase 4
THPO: Thrombopoietin
DECR1: 2,4-dienoyl-CoA reductase 1
ITGB1BP2: Integrin Subunit Beta 1 Binding Protein 2
NEMO: Nuclear factor κB (NF-κB) essential modulator
HSP27: Heat shock protein-27
IL27: Interleukin 27
HSP: Heat shock proteins
