# Supplementary material for: Sleeping for two: a cross-sectional study on associations between objectively measured sleep during early to mid-pregnancy and maternal and fetal outcomes and inflammatory biomarker profiles
Source: BMC Pregnancy Childbirth. 2025 May 5;25:533. doi: 10.1186/s12884-025-07634-9 (PMC12054240; doi:10.1186/s12884-025-07634-9)
Supplement: Supplementary file 1 — Supplementary Material 1 [file 12884_2025_7634_MOESM1_ESM.docx]

**Supplementary Table 1:** Group presentation of the remaining inflammatory markers that showed no significant differences

|  | **Good sleep**  **N=** 728 (50.4%) | | **Intermediate sleep**  **N=** 471 (32.6%) | | **Poor sleep**  **N=** 245 (17.0%) | |  |
| --- | --- | --- | --- | --- | --- | --- | --- |
| **Variable** *N=407* | **N** | **Mean±SD** | **N** | **Mean±SD** | **N** | **Mean±SD** | **P-value** a |
| BMP6 | 196 | 3.73 ± 0.82 | 146 | 3.69 ± 0.78 | 65 | 3.71 ± 0.79 | 0.890 |
| ANGPT1 | 196 | 7.70 ± 0.89 | 146 | 7.70 ± 0.88 | 65 | 7.75 ± 0.85 | 0.919 |
| ADM | 196 | 7.37 ± 0.36 | 146 | 7.36 ± 0.37 | 65 | 7.46 ± 0.37 | 0.128 |
| SLAM47 | 196 | 3.02 ± 0.65 | 146 | 3.02 ± 0.54 | 65 | 2.90 ± 0.49 | 0.304 |
| PGF | 196 | 9.84 ± 0.68 | 146 | 9.94 ± 0.71 | 65 | 9.96 ± 0.77 | 0.326 |
| ADAMTS13 | 196 | 6.33 ± 0.16 | 146 | 6.33 ± 0.16 | 65 | 6.36 ± 0.19 | 0.454 |
| BOC | 196 | 3.76 ± 0.25 | 146 | 3.75 ± 0.26 | 65 | 3.77 ± 0.26 | 0.864 |
| IL4RA | 196 | 2.15 ± 0.28 | 146 | 2.18 ± 0.30 | 65 | 2.22 ± 0.28 | 0.256 |
| IL1ra | 196 | 4.34 ± 0.57 | 146 | 4.39 ± 0.52 | 65 | 4.49 ± 0.65 | 0.168 |
| IL6 | 196 | 2.31 ± 0.60 | 146 | 2.29 ± 0.78 | 65 | 2.36 ± 0.54 | 0.807 |
| TNFRSF10A | 196 | 3.347 ± 0.33 | 146 | 3.36 ± 0.31 | 65 | 3.42 ± 0.29 | 0.497 |
| IDUA | 196 | 5.95 ± 0.47 | 146 | 5.84 ± 0.46 | 65 | 6.01 ± 0.44 | **0.028** |
| TNFRSF11A | 196 | 5.24 ± 0.35 | 146 | 5.23 ± 0.33 | 65 | 5.30 ± 0.35 | 0.387 |
| PAR1 | 196 | 8.34 ± 0.44 | 146 | 8.24 ± 0.35 | 65 | 8.28 ± 0.43 | 0.065 |
| TRAILR2 | 196 | 5.04 ± 0.35 | 146 | 5.03 ± 0.32 | 65 | 5.07 ± 0.27 | 0.729 |
| PRSS27 | 196 | 8.37 ± 0.44 | 146 | 8.39 ± 0.46 | 65 | 8.45 ± 0.51 | 0.518 |
| TIE2 | 196 | 6.88 ± 0.25 | 146 | 6.86 ± 0.24 | 65 | 6.91 ± 0.30 | 0.438 |
| TF | 196 | 4.33 ± 0.27 | 146 | 4.32 ± 0.27 | 65 | 4.34 ± 0.24 | 0.859 |
| IL1RL2 | 196 | 3.50 ± 0.43 | 146 | 3.44 ± 0.49 | 65 | 3.43 ± 0.47 | 0.406 |
| PDGFsubunitB | 196 | 8.45 ± 0.90 | 146 | 8.39 ± 0.86 | 65 | 8.53 ± 0.75 | 0.543 |
| IL17D | 196 | 1.76 ± 0.28 | 146 | 1.76 ± 0.30 | 65 | 1.74 ± 0.28 | 0.901 |
| CXCL1 | 196 | 8.72 ± 0.90 | 146 | 8.65 ± 0.84 | 65 | 8.81 ± 0.87 | 0.462 |
| LOX1 | 196 | 6.78 ± 0.41 | 146 | 6.80 ± 0.42 | 65 | 6.91 ± 0.42 | 0.087 |
| Gal9 | 196 | 7.15 ± 0.31 | 146 | 7.13 ± 0.35 | 65 | 7.18 ± 0.32 | 0.620 |
| GIF | 196 | 6.87 ± 0.74 | 146 | 6.94 ± 0.66 | 65 | 6.91 ± 0.91 | 0.673 |
| SCF | 196 | 8.40 ± 0.36 | 146 | 8.34 ± 0.45 | 65 | 8.43 ± 0.37 | 0.204 |
| IL18 | 196 | 8.36 ± 0.56 | 146 | 8.37 ± 0.51 | 65 | 8.35 ± 0.53 | 0.965 |
| FGF21 | 196 | 4.50 ± 1.51 | 146 | 4.67 ± 1.57 | 65 | 4.59 ± 1.33 | 0.586 |
| PlgR | 196 | 6.26 ± 0.13 | 146 | 6.25 ± 0.13 | 65 | 6.25 ± 0.13 | 0.910 |
| RAGE | 196 | 12.78 ± 0.38 | 146 | 12.73 ± 0.40 | 65 | 12.86 ± 0.35 | 0.075 |
| SOD2 | 196 | 9.38 ± 0.13 | 146 | 9.39 ± 0.14 | 65 | 9.40 ± 0.13 | 0.767 |
| CTRC | 196 | 10.24 ± 0.63 | 146 | 10.16 ± 0.69 | 65 | 10.04 ± 0.58 | 0.100 |
| FGF23 | 196 | 3.70 ± 0.58 | 146 | 3.68 ± 0.52 | 65 | 3.74 ± 0.66 | 0.778 |
| SPON2 | 196 | 8.22 ± 0.17 | 146 | 8.20 ± 0.18 | 65 | 8.23 ± 0.18 | 0.521 |
| GH | 196 | 8.71 ± 0.52 | 146 | 8.66 ± 0.44 | 65 | 8.61 ± 0.55 | 0.332 |
| FS | 196 | 12.71 ± 0.37 | 146 | 12.69 ± 0.49 | 65 | 12.80 ± 0.44 | 0.180 |
| GLO1 | 196 | 6.45 ± 0.60 | 146 | 6.43 ± 0.52 | 65 | 6.52 ± 0.46 | 0.566 |
| CD84 | 196 | 4.12 ± 0.40 | 146 | 4.07 ± 0.30 | 65 | 4.08 ± 0.35 | 0.405 |
| PAPPA | 196 | 10.52 ± 0.25 | 146 | 10.55 ± 0.24 | 65 | 10.50 ± 0.26 | 0.299 |
| SERPINA12 | 196 | 4.17 ± 0.99 | 146 | 4.18 ± 0.94 | 65 | 4.10 ± 1.03 | 0.853 |
| REN | 196 | 7.68 ± 0.47 | 146 | 7.68 ± 0.45 | 65 | 7.68 ± 0.44 | 0.999 |
| MERTK | 196 | 5.86 ± 0.39 | 146 | 5.82 ± 0.33 | 65 | 5.89 ± 0.39 | 0.370 |
| KIM1 | 196 | 6.27 ± 0.52 | 146 | 6.22 ± 0.54 | 65 | 6.23 ± 0.57 | 0.657 |
| THBS2 | 196 | 5.57 ± 0.17 | 146 | 5.54 ± 0.17 | 65 | 5.55 ± 0.17 | 0.187 |
| TM | 196 | 10.10 ± 0.27 | 146 | 10.09 ± 0.28 | 65 | 10.11 ± 0.27 | 0.832 |
| VSIG2 | 196 | 3.51 ± 0.38 | 146 | 3.60 ± 0.43 | 65 | 3.61 ± 0.48 | 0.090 |
| AMBP | 196 | 7.41 ± 0.16 | 146 | 7.41 ± 0.15 | 65 | 7.45 ± 0.17 | 0.206 |
| PRELP | 196 | 7.81 ± 0.18 | 146 | 7.80 ± 0.18 | 65 | 7.84 ± 0.16 | 0.390 |
| HO1 | 196 | 10.88 ± 0.43 | 146 | 10.86 ± 0.42 | 65 | 10.82 ± 0.47 | 0.624 |
| XCL1 | 196 | 4.50 ± 0.50 | 146 | 4.43 ± 0.45 | 65 | 4.41 ± 0.49 | 0.268 |
| IL16 | 196 | 5.63 ± 0.43 | 146 | 5.61 ± 0.42 | 65 | 5.71 ± 0.48 | 0.301 |
| SORT1 | 196 | 8.17 ± 0.28 | 146 | 8.11 ± 0.23 | 65 | 8.16 ± 0.25 | 0.081 |
| CEACAM8 | 196 | 3.88 ± 0.51 | 146 | 3.96 ± 0.46 | 65 | 4.03 ± 0.50 | 0.056 |
| PTX3 | 196 | 4.21 ± 0.32 | 146 | 4.20 ± 0.35 | 65 | 4.27 ± 0.31 | 0.393 |
| PSGL1 | 196 | 3.71 ± 0.22 | 146 | 3.69 ± 0.22 | 65 | 3.71 ± 0.21 | 0.589 |
| CCL17 | 196 | 6.60 ± 0.91 | 146 | 6.41 ± 0.83 | 65 | 6.61 ± 1.01 | 0.118 |
| CCL3 | 196 | 5.35 ± 0.51 | 146 | 5.29 ± 0.50 | 65 | 5.33 ± 0.65 | 0.591 |
| MMP7 | 196 | 10.09 ± 0.42 | 146 | 10.06 ± 0.41 | 65 | 10.14 ± 0.40 | 0.449 |
| IgGFcreceptorIIb | 196 | 3.04 ± 0.77 | 146 | 2.96 ± 0.86 | 65 | 3.09 ± 0.77 | 0.484 |
| DCN | 196 | 4.11 ± 0.26 | 146 | 4.09 ± 0.25 | 65 | 4.11 ± 0.27 | 0.437 |
| Dkk1 | 196 | 8.53 ± 0.47 | 146 | 8.48 ± 0.43 | 65 | 8.52 ± 0.45 | 0.634 |
| LPL | 196 | 9.40 ± 0.46 | 146 | 9.43 ± 0.45 | 65 | 9.44 ± 0.49 | 0.767 |
| PRSS8 | 196 | 8.66 ± 0.24 | 146 | 8.68 ± 0.29 | 65 | 8.75 ± 0.25 | 0.066 |
| AGRP | 196 | 4.87 ± 0.39 | 146 | 4.89 ± 0.41 | 65 | 4.98 ± 0.53 | 0.211 |
| HBEGF | 196 | 5.19 ± 0.49 | 146 | 5.13 ± 0.42 | 65 | 5.18 ± 0.42 | 0.512 |
| GDF2 | 196 | 9.40 ± 0.47 | 146 | 9.29 ± 0.44 | 65 | 9.44 ± 0.43 | **0.022** |
| FABP2 | 196 | 7.42 ± 0.83 | 146 | 7.45 ± 0.90 | 65 | 7.53 ± 0.99 | 0.671 |
| MARCO | 196 | 6.21 ± 0.28 | 146 | 6.22 ± 0.23 | 65 | 6.20 ± 0.24 | 0.892 |
| @GT | 196 | 0.95 ± 0.51 | 146 | 0.96 ± 0.63 | 65 | 0.98 ± 0.56 | 0.948 |
| BNP | 196 | 1.15 ± 0.58 | 146 | 1.21 ± 0.62 | 65 | 1.27 ± 0.75 | 0.414 |
| MMP12 | 196 | 6.99 ± 0.62 | 146 | 6.94 ± 0.70 | 65 | 7.00 ± 0.68 | 0.785 |
| ACE2 | 196 | 3.81 ± 0.54 | 146 | 3.77 ± 0.55 | 65 | 3.88 ± 0.56 | 0.423 |
| PDL2 | 196 | 3.26 ± 0.30 | 146 | 3.29 ± 0.36 | 65 | 3.35 ± 0.29 | 0.164 |
| CTSL1 | 196 | 5.99 ± 0.28 | 146 | 5.97 ± 0.28 | 65 | 6.01 ± 0.30 | 0.701 |
| hOSCAR | 196 | 10.33 ± 0.22 | 146 | 10.32 ± 0.24 | 65 | 10.31 ± 0.22 | 0.863 |
| TNFRSF13B | 196 | 9.52 ± 0.35 | 146 | 9.53 ± 0.34 | 65 | 9.51 ± 0.36 | 0.916 |
| TGM2 | 196 | 8.16 ± 0.44 | 146 | 8.15 ± 0.47 | 65 | 8.20 ± 0.45 | 0.744 |
| LEP | 196 | 7.45 ± 0.70 | 146 | 7.41 ± 0.76 | 65 | 7.49 ± 0.78 | 0.764 |
| CA5A | 196 | 2.02 ± 0.91 | 146 | 2.03 ± 0.93 | 65 | 2.09 ± 0.82 | 0.880 |
| CD4 | 196 | 4.48 ± 0.26 | 146 | 4.46 ± 0.33 | 65 | 4.50 ± 0.28 | 0.745 |
| VEGFD | 196 | 7.63 ± 0.39 | 146 | 7.56 ±0.46 | 65 | 7.62 ± 0.39 | 0.344 |
| PARP1 | 196 | 1.60 ± 0.44 | 146 | 1.59 ± 0.39 | 65 | 1.55 ± 0.41 | 0.660 |
| HAOX1 | 196 | 4.59 ± 1.59 | 146 | 4.50 ± 1.65 | 65 | 4.50 ± 1.58 | 0.836 |
